# The Role of Renal PLA2R Staining Combined with Serum PLA2R Antibody in Membranous Nephropathy Risk Stratification

**DOI:** 10.3390/jcm13010068

**Published:** 2023-12-22

**Authors:** Xiaofan Hu, Xinlu Wang, Xialian Yu, Liyan Ni, Chenni Gao, Xiaoxia Pan, Hong Ren, Jing Xu, Jun Ma

**Affiliations:** Department of Nephrology, Institute of Nephrology, Shanghai Ruijin Hospital, School of Medicine, Shanghai Jiao Tong University, Shanghai 200025, China; hxf12268@rjh.com.cn (X.H.); wxl01e21@rjh.com.cn (X.W.); yxl40653@rjh.com.cn (X.Y.); nly40534@rjh.com.cn (L.N.); gcn11933@rjh.com.cn (C.G.); panxiaoxia@medmail.com.cn (X.P.); rh10492@rjh.com.cn (H.R.)

**Keywords:** PMN, PLA2R antibody, PLA2R staining, renal prognosis

## Abstract

Background: This study aimed to examine the clinicopathological profiles and prognosis of membranous nephropathy in different subtypes classified by serum PLA2R antibody (SAb) and glomerular PLA2R antigen staining (GAg). Methods: A total of 372 biopsy-proven membranous nephropathy (MN) cases, unrelated to lupus, with urine protein > 2 g/24 h and eGFR > 25 mL/min/1.73 m^2^ were included and categorized into four groups according to the presence or absence of PLA2R antibody and glomerular PLA2R antigen staining. Clinical profiles were compared among four subtypes. Treatment response and renal outcomes were compared among four groups with primary MN. Cox and logistic regression models were used to examine the association between time-to-renal progression and early remission within 6 months in the four subgroups with primary MN. Results: MN patients who were SAb−/GAg+ presented with a more severe disease onset, whereas those who were SAb−/GAg− had a mild clinical manifestation with a higher prevalence of MN-associated secondary causes. During a median follow-up of 79.2 months (IQR: 48.70–97.40), SAb+/GAg− was identified as an independent risk factor for renal progression [HR: 9.17, 95% CI: 2.26–37.16, *p* < 0.01] and early remission [OR: 0.06, 95% CI: 0.01–0.56, *p* = 0.01] in primary MN. Additionally, SAb−/GAg− with primary MN showed an independent association with spontaneous remission after adjusting for age, sex, baseline proteinuria, and eGFR (Before adjustment: OR: 8.33, 95% CI: 1.89–36.76, *p* = 0.0; after adjustment: OR: 12.25, 95% CI: 2.48–60.53, *p* < 0.01). Conclusion: Our findings indicated that SAb+/GAg−MN patients exhibited a more severe disease onset and had a poorer prognosis, necessitating an aggressive treatment approach. On the other hand, in the SAb−/GAg− group, the elimination of secondary causes should be considered, and a watchful waiting approach may be appropriate.

## 1. Introduction

Primary Membranous Nephropathy (PMN) is an organ-specific autoimmune disease characterized by thickening of the glomerular basal membrane (GBM) and deposition of subepithelial immune complexes. It is one of the most common forms of adult-onset primary glomerulonephritis (PGN), accounting for 11.36~23.94% of PGN cases [1,2]. A dramatic increase in PMN in China has been reported in recent years [3], possibly attributed to rising air pollution levels.

New discoveries of target antigens [4,5,6], along with genetic susceptibility loci [7,8,9], uncovered novel pathogenic mechanisms underlying primary MN. Notably, PLA2R antigen [4] was identified as the most common podocyte antigen in PMN. Approximately 70–80% [4,10] patients were considered PLA2R-associated PMN, based on the presence of circulating PLA2R antibodies in serum (SAb) and/or detection of PLA2R antigen in renal biopsies (GAg). SAb and GAg can be used to classify MN patients into four different subgroups: patients with negative serum PLA2R antibody and negative glomerular PLA2R staining (SAb−/GAg−), patients with positive serum PLA2R antibody and negative glomerular PLA2R staining (SAb+/GAg−), patients with positive serum PLA2R antibody and positive glomerular PLA2R staining (SAb+/GAg+) and patients with negative serum PLA2R antibody and positive glomerular PLA2R staining (SAb−/GAg+). However, there are limited data available on the distinct characteristics of the four subgroups based on SAb and GAg. Most of the published studies mainly focused on SAb−/GAg− MN patients, an entity of MN that shared a different pathogenesis from the other three subgroups. In a study by Hanset et al. [11], 177 MN cases unrelated to lupus were divided into three types based on their podocyte antigen staining, including PLA2R, THSD7A, and NELL-1. The study suggested that the subgroup with PLA2R SAb−/GAg− shared a similar renal disease course with PLA2R-related PMN, while those with PLA2R SAb−/GAg− were more prone to developing adverse events such as cancer and death during follow-up. Yet in a study by Wang et al. [12], SAb−/GAg− patients appeared more likely to achieve remission than those with PLA2R-related PMN, with no evidence of higher tumor rates. These published studies have produced conflicting results, highlighting the need for further clinical investigations.

In addition, previous studies [13] demonstrated that apart from PLA2R SAb−/GAg− MN, the other three subtypes may represent distinct phases of the MN disease course. PLA2R SAb+/GAg− PMN, accounting for 1.3–13.8% PMN [14,15,16], was considered as an early-stage phase of PMN. However, this notion has been challenged by a recently published study [14]. In a cohort of 130 PLA2R SAb+ MNs, Lou et al. [14] suggested that SAb+/GAg− was a distinct MN subtype characterized by more severe disease and worse renal prognosis. As the study sample was limited, the conclusion should be further tested in a large-scale study. In addition, the aforementioned studies did not investigate whether specific treatment strategies may be more effective for certain subgroups, highlighting the need for future research in this area.

To address the existing research gap and provide new insights into risk stratification for patients with primary membranous nephropathy (PMN), we conducted a comprehensive analysis of a substantial retrospective cohort comprising 372 MN patients, with a median follow-up time of 79 months to investigate the clinical characteristics, renal prognosis, and treatment response among various subtypes based on renal PLA2R antigen staining and serum PLA2R ab test, aiming to provide new evidence for enhanced risk stratification in PMN.

## 2. Materials and Methods

### 2.1. Participants and Study Design

**Figure 1:**
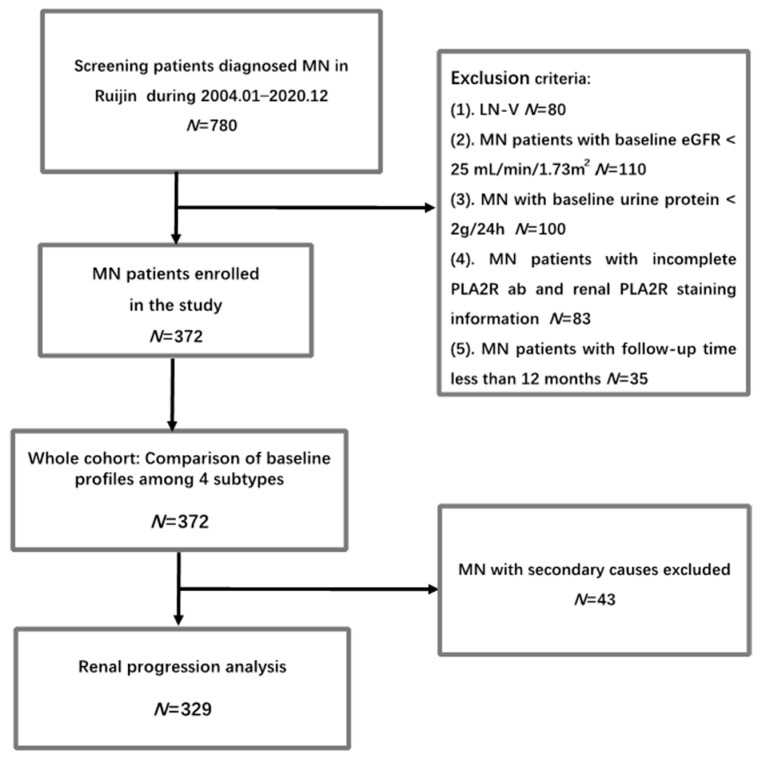
Flowchart of the study.

This retrospective study involved 372 patients diagnosed with membranous nephropathy in Shanghai Ruijin Hospital, Shanghai Jiao Tong University School of Medicine, between January 2004 and December 2020. The inclusion criteria are listed below: 1. Patients aged between 14 and 85 years; 2. The diagnosis of MN was confirmed by kidney biopsy; 3. Availability of both PLA2R1 staining in kidney tissues and Elisa test results for serum PLA2R antibodies at baseline; 4. Baseline urine protein excretion exceeding 2 g/24 h and an estimated glomerular filtration rate (eGFR) greater than 25 mL/min/1.73m^2^ (In previous studies investigating the prognostic role of either PLA2R antibody or glomerular PLA2R staining in MN [11,14], the inclusion criteria did not establish specific standards for urine protein or eGFR levels. However, these studies typically included patients with a minimum urine protein level of 2.6 g/24 h and an eGFR above 30. Thus, we established a criterion of 2 g/24 h for urine protein inclusion. Additionally, we aimed to investigate renal prognosis and treatment outcomes, which is why we excluded patients with severe renal insufficiency and included those with an eGFR greater than 25 mL/min × 1.73m^2^, similar to the inclusion criteria used in the DAPA-CKD trial [17]. By setting these criteria, we can better focus on patients who are likely to benefit from our study’s interventions and obtain more meaningful results); 5. Minimum follow-up duration of at least 12 months in the absence of renal progression or death; and 6. Patients consented to participate in the study. Exclusion criteria comprised MN secondary to systemic lupus erythematosus (SLE) and MN complicated with other glomerular diseases. The patients received treatment guided by the Kidney Disease: Improving Global Outcomes (KDIGO) guidelines. The choice of immunosuppressive regimen was proposed according to the KDIGO guidelines and determined following discussions with the patients. Patients were censored at the endpoint if they were lost to follow-up or in the event of death. All patients provided written informed consent to participate in the study (Figure 1).

### 2.2. Data Collection

Demographic features and clinical data of the patients at the time of diagnosis were collected from their medical records. eGFR was calculated by the CKD-EPI eGFR formula [18]. The baseline PLA2R ab levels were tested by ELISA using a standard protocol as recommended by the manufacturer (Euroimmun, Lübeck, Germany), and serum PLA2R ab level higher than 20 RU/mL was considered positive. The renal biopsy was independently examined and scored by two renal pathologists. Glomerular PLA2R1 staining was performed by an indirect immunofluorescence test with an anti-PLA2R1 antibody (Atlas Antibodies AB, Stockholm, Sweden). The presence of granular capillary loop staining in the glomeruli was defined as positive. And self-control was used as a negative control. Patients with negative PLA2R staining in immunofluorescence were reexamined and confirmed by IHC. All patients were followed up regularly, and information on urine protein, serum albumin, and serum creatinine was recorded during each follow-up visit. Complete remission was defined as proteinuria < 0.3 g/24 h and absence of deterioration of renal function. Partial remission was defined as proteinuria < 3.5 g/24 h and a urine protein reduction by at least 50% compared to baseline with stable renal function. Remission was classified as either complete or partial remission. Spontaneous remission was defined as remission without application of steroids and immunosuppressive therapy. Renal progression was defined as a decrease in eGFR by at least 30% compared to baseline eGFR. 

### 2.3. Statistical Analysis

Continuous variables with a skewed distribution were presented as the median (interquartile range). They were compared by Mann–Whitney U test, while customarily distributed variables were displayed as mean ± SD and compared by *t*-test. Categorical variables were compared with a chi-squared test. The Kaplan–Meier survival curves were applied to compare the different renal outcomes among 4 subgroups. The Cox regression model was employed to assess the association between each predictor and time-to-renal progression. The logistic regression model was applied to examine the association between each predictor and remission within 6 months. *p* value < 0.05 was considered significant. Statistical analysis was performed with SPSS software (version 21.0) and R software (CRAN, R version 4.1.2).

## 3. Results

### 3.1. Baseline Characteristics of the Whole Cohort and Comparison of Baseline Profiles among Different Subtypes Based on PLA2R ab and Glomerular PLA2R Staining in PMN

In total, 372 MN patients were enrolled in the study. The median age was 56 (interquartile range, IQR: 43.75–64) years old, with a male predominance (male: female ratio: 1:0.57). The median eGFR at the time of biopsy was 99.13 (IQR: 78.61–113.92) mL/min/1.73 m^2^, urine protein was 5.71 (IQR: 4.11–8.25) g/24 h, serum albumin was 21 (IQR: 17–26) g/L and serum PLA2R ab level was 28.22 (IQR: 3.50–96.38) RU/mL. Among the patients, 45.70% had hypertension, 13.98% had diabetes mellitus, and 11.56% may have had secondary causes such as HBV/HCV infection, solid tumor, psoriasis, or monoclonal gammopathy of renal significance (MGRS). A total of 20.70% (*n* = 77) patients were diagnosed as stage I MN, 66.13% (*n* = 246) with stage II MN, and 13.17% (*n* = 49) with Stage III/IV MN. Moderate or severe tubulo-interstitial lesions were observed in 2.15% (*n* = 8) of the patients. In total, 83.60% of MN patients were PLA2R1 positive in renal tissues (Table 1).

Based on the status of serum PLA2R ab (SAb) and Glomerular PLA2R antigen staining in (GAg), MN patients were then categorized into four subgroups: SAb−/GAg− (negative serum PLA2R antibody and negative glomerular PLA2R staining, 14.52%, *n* = 54), SAb +/GAg− (positive serum PLA2R antibody and negative glomerular PLA2R staining, 1.89%, *n* = 7), SAb+/GAg+ (positive serum PLA2R antibody and positive glomerular PLA2R staining, 54.03%, *n* = 201) and SAb−/GAg+ (negative serum PLA2R antibody and positive glomerular PLA2R staining, 29.57%, *n* = 110). Compared to the other three subgroups, SAb−/GAg− MN patients exhibited a milder disease onset with less urine protein (median urine protein at biopsy SAb−/GAg− vs. SAb+/GAg− vs. SAb+/GAg+ vs. SAb−/GAg+: 5.39 vs. 6.84 vs. 6.38 vs. 5.09 g/24 h, *p* = 0.01), higher serum albumin (median serum albumin at biopsy SAb−/GAg− vs. SAb+/GAg− vs. SAb+/GAg+ vs. SAb−/GAg+: 23.5 vs. 14.0 vs. 21.0 vs. 23.5 g/L, *p* < 0.01) and a lower prevalence of hypertension (SAb−/GAg− vs. SAb+/GAg− vs. SAb+/GAg+ vs. SAb−/GAg+: 22.22% vs. 42.86% vs. 50.25% vs. 49.09%, *p* < 0.01) (Table 1). Conversely, SAb+/GAg− presented with higher levels of urine protein, lower albumin, and higher PLA2R ab levels at baseline. When screening for secondary causes, a higher proportion of SAb−/GAg− MN patients appeared to have secondary causes (SAb−/GAg− vs. SAb+/GAg− vs. SAb+/GAg+ vs. SAb−/GAg+: 24.07% vs. 14.29% vs. 9.45% vs. 9.09%, *p* = 0.02). (Table 1) 

### 3.2. Comparison of Renal Prognosis among Different Subtypes Based on PLA2R ab and Glomerular PLA2R Staining in PMN

**Figure 2:**
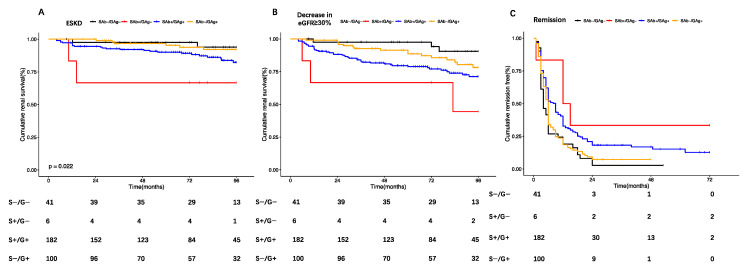
Comparison of Kaplan–Meier curves among 4 groups for different renal outcomes: (**A**). ESRD: red lines: SAb+/GAg−; black lines: SAb−/GAg−; orange: SAb+/GAg+; blue: SAb−/GAg+; SAb−/GAg−; as reference: SAb+/GAg−: HR: 6.49, 95% CI: 1.08–39.08, *p* = 0.04; SAb−/GAg+: HR:0.83, 95% CI: 0.21–3.33, *p* = 0.80; SAb+/GAg+: HR: 2.34, 95% CI: 0.71–7.72, *p* = 0.16. (**B**) Renal progression: red lines: SAb+/GAg−; black lines: SAb−/GAg−; orange: SAb+/GAg+; blue: SAb−/GAg+; SAb−/GAg−; as reference: SAb+/GAg−: HR: 9.13, 95% CI: 2.28–36.62, *p* < 0.01; SAb−/G+: HR: 1.92, 95% CI: 0.65–5.67, *p* = 0.24; SAb+/GAg+: HR: 3.26, 95% CI: 1.17–9.07, *p* = 0.02. (**C**) Remission: red lines: SAb+/GAg−; black lines: SAb−/GAg−; orange: SAb+/GAg+; blue: SAb−/GAg+; SAb−/GAg−; as reference: SAb+/GAg−: HR: 0.27, 95% CI: 0.10–0.76, *p* = 0.01; SAb−/GAg+: HR: 0.94, 95% CI: 0.65–1.36, *p* = 0.73; SAb+/GAg+: HR: 0.63, 95% CI: 0.44–0.89, *p* = 0.01.

Patients with primary PMN were included for further analysis on the comparison of renal prognosis among different subtypes based on PLA2R ab and glomerular PLA2R staining. During a median follow-up time of 79.2 (IQR: 48.70–97.40) months, 73 (22.19%) patients developed renal progression, with 11.55% (*n* = 38) developing end-stage renal disease (ESRD). Early remission was achieved in 55.62% of the patients (*n* = 183) within 6 months and in 85.41% (*n* = 218) by the end of 24 months. Thirteen patients died. The main causes of death were infection and cardiovascular disease. 3.65% of the patients (*n* = 12) developed tumors, and 7.60% (*n* = 25) experienced thrombosis events. Among four subtypes, SAb−/GAg− MN patients demonstrated a more favorable renal outcome, with a higher rate of remission achieved within 6 months (SAb−/GAg− vs. SAb+/GAg− vs. SAb+/GAg+ vs. SAb−/GAg+: 73.17% vs. 16.67% vs. 47.80% vs. 65%, *p* < 0.01), a lower incidence of renal progression (SAb−/GAg− vs. SAb+/GAg− vs. SAb+/GAg+ vs. SAb−/GAg+: 9.76% vs. 66.67% vs. 25.82% vs. 18.00%, *p* < 0.01) and a lower occurrence of ESRD. (SAb−/GAg− vs. SAb+/GAg− vs. SAb+/GAg+ vs. SAb−/GAg+: 7.32% vs. 33.33% vs. 14.84% vs. 6%, *p* = 0.04), while SAb+/GAg− MN patients presented with a higher incidence of renal progression and fewer early remissions within 6 months. There was no significant difference among four groups in death, thrombosis, and tumor development during the follow-up time (Table 1 and Figure 2). 

### 3.3. Predictors of Early Remission and Renal Progression in PMN

Via univariate Cox regression analysis, we identified several factors that were associated with renal progression in primary MN patients, including age, urine protein, eGFR, hypertension, diabetes, use of immunosuppressive therapy, tubulo-interstitial lesions ≥ 25%, SAb+/GAg−, and SAb+/GAg+. However, in multivariate analysis, only SAb+/GAg− [HR: 9.17, 95% CI: 2.26–37.16, *p* < 0.01], age [HR: 1.06, 95% CI: 1.03–1.08, *p* < 0.01], and diabetes [HR:2.35, 95% CI: 1.35–4.09, *p* < 0.01] were found to be independent risk factors for renal progression. (Table 2 and Figure 2b). Regarding early remission, our findings revealed that urine protein (OR: 0.92, 95% CI: 0.85–0.98, *p* = 0.02), diabetes mellitus (OR: 0.47, 95% CI: 0.24–0.94, *p* = 0.03), immunosuppressive agents (OR: 3.19, 95% CI: 1.78–5.73, *p* < 0.01), SAb+/GAg− (OR: 0.06, 95% CI: 0.01–0.56, *p* = 0.01), and SAb+/GAg+ (OR: 0.30,95% CI: 0.14–0.66, *p* < 0.01) were independently related to early remission in multivariate analysis. It is noteworthy that patients with SAb+/GAg− status were more likely to experience renal progression and to remain unresponsive to therapy within 6 months (Table 3).

### 3.4. Comparison of Response to Immunosuppressive Therapy among Different Subtypes

Subsequently, we categorized patients with primary PMN into different treatment strategies to evaluate the response to immunosuppressive therapy across various subtypes. Among patients treated with ACEI/ARB, the SAb−/GAg− group showed a higher likelihood of achieving spontaneous remission compared to the other subgroups (SAb−/GAg− vs. SAb+/GAg− vs. SAb+/GAg+ vs. SAb−/GAg+: 76.92% vs. 0% vs. 28.57% vs. 51.43%, *p* = 0.01). To further validate the association, logistic regression models were performed with sequential adjustment. Our analysis revealed that SAb−/GAg− was strongly associated with spontaneous remission, both before adjustment (OR: 8.33, 95% CI: 1.89–36.76, *p* = 0.01) and after adjustment for age, sex, baseline proteinuria, and eGFR (OR: 12.25, 95% CI: 2.48–60.53, *p* < 0.01). (Table 4). In patients treated with cyclosporine, the SAb−/GAg+ group exhibited a higher remission rate within six months compared to the other subgroups. (SAb−/GAg− vs. SAb+/GAg− vs. SAb+/GAg+ vs. SAb−/GAg+: 75% vs. 50% vs. 57.89% vs. 96.77%, *p* < 0.01). SAb−/GAg+ was identified as an independent predictor of early remission induced by cyclosporine, both before adjustment (OR: 21.82, 95% CI: 2.83–168.42, *p* < 0.01) and after adjustment for age, sex, baseline proteinuria, and eGFR (OR: 16.77, 95% CI: 2.10–134.14, *p* = 0.01). There was no significant difference in the remission rate induced by cyclophosphamide among the different subgroups (Table 4).

## 4. Discussion

The identification of PLA2R ab in PMN has led to a significant shift in the diagnosis and monitoring of patients with PMN [4,19]. Currently, PLA2R antibody is recommended as one of the most important criteria for risk stratification in evaluating PMN [19]. However, the clinical significance of glomerular PLA2R staining combined with serum PLA2R antibodies has seldom been discussed. Therefore, we conducted a study involving 372 MN patients with a median follow-up time of 79 months to investigate the characteristics of four subtypes based on serum PLA2R antibodies and glomerular PLA2R staining. Our findings revealed that MN with PLA2R SAb−/GAg− patients were more likely to be associated with secondary causes and had a better renal prognosis, with a higher occurrence of spontaneous remission. In contrast, both SAb+/GAg− and SAb+/GAg+ subtypes showed a more severe onset and worse renal outcome, particularly SAb+/GAg−. Consequently, our study suggests that a more aggressive treatment strategy should be adopted for SAb+/GAg− patients, while SAb−/GAg− patients should undergo the elimination of secondary causes and may benefit from a wait-and-see approach. Our study highlights the importance of considering both serum PLA2R antibodies and glomerular PLA2R staining in evaluating the pathogenesis and prognosis of MN. These findings have significant implications for better risk stratification and tailored therapeutic approaches for MN patients.

With the advent of laser microdissection and mass spectrometry, new antigens in podocytes were consecutively identified in MN patients. Among these, PLA2R was found to be the predominant antigen, accounting for 70–80% of MN cases, followed by NELL-1 8%, THSD7A 3%, and Semaphorin 3B 1% [6,20,21]. Several studies suggested that SAb−/GAg− subtypes may be more likely associated with secondary causes, such as tumors and autoimmune diseases [21,22]. In our study, we confirmed the association and found that SAb−/GAg− patients exhibited a better renal prognosis, with more rapid remissions, lower incidence of end-stage renal disease (ESRD), and higher occurrence of spontaneous remissions. These findings are partially aligned with Hanset et al.’s study [11] involving 177 MN patients, which reported a tendency toward higher 5-year remission rates and lower spontaneous remission rates in patients with PLA2R-related MN. Considering the variation in treatment regimens among different groups, with more PLA2R-related MN patients receiving multiple immunosuppressive therapies, this may explain the higher remission rates observed in PLA2R-related MN. In Wang et al. [12]’s study with 832 MN patients enrolled, they found that non-PLA2R-related MN patients were more likely to achieve remission, consistent with our study. Taken together with previous studies, our findings suggest that SAb−/GAg− MN patients are more prone to experiencing remission.

SAb+/GAg− MN represented a special entity of PLA2R-related MN, characterized by the presence of circulating PLA2R ab in serum but negative PLA2R1 staining in renal tissues. In our study, the prevalence of SAb+/GAg− was 1.89%, consistent with previous reports [15,16,23]. The underlying mechanism of SAb+/GAg− remained unknown. One study [13] speculated that it may be an early form of MN, while Luo et al. [14] suggested a potential association with PLA2R epitope spreading. Our study revealed that SAb+/GAg− presented a more severe onset with higher baseline urine protein and PLA2R antibody levels. Furthermore, these patients demonstrated a higher risk of renal progression, including end-stage renal disease (ESRD), during follow-up. These findings confirmed Luo et al.’s [14] results with a longer follow-up duration. Additionally, we found that SAb+/GAg− was independently associated with early remission and renal progression, both before and after adjustment, indicating a potential need for more aggressive therapy in this subtype.

In comparison to the previously mentioned subtypes, SAb−/GAg+ may present with moderate disease onset and renal prognosis. SAb−/GAg+, characterized by low levels of PLA2R antibodies in serum, was considered not to be in active immunological status and exhibited a better renal prognosis with a lower incidence of ESRD. Conversely, SAb+/GAg+ patients show a lower rate of early remission and a higher rate of renal progression, similar to SAb+/GAg−, consistent with previous studies [16]. 

Our study has several limitations. Firstly, as a single-center study, the generalizability of our conclusions may be limited, and further validation with a more ethnically diverse population of MN patients is needed. Secondly, we tried to incur more valuable patients to our study based on available data to complete our research, and subgroup analysis according to their eGFR level and risk stratification would be carried out in further study when more patients with MN are enrolled. Thirdly, due to the retrospective nature of our study, treatment strategies varied among patients. Although we adjusted for multiple factors related to ESRD and remission to test the association between treatment response and GAg and SAb, a prospective cohort study would be beneficial in confirming our findings.

## 5. Conclusions

Our findings indicated that SAb+/GAg− MN patients exhibited a more severe disease onset and had a poorer prognosis, necessitating an aggressive treatment approach. On the other hand, in the SAb−/GAg− group, the elimination of secondary causes should be considered, and a watchful waiting approach may be appropriate. 

## Figures and Tables

**Table 1:** Comparison of clinicopathological profiles and prognosis among four subgroups based on glomerular PLA2R antigen staining and serum PLA2R ab test.

	All	SAb−/GAg−	SAb+/GAg−	SAb+/GAg+	SAb−/GAg+	*p* Value
No (%)	372	54 (14.52)	7 (1.89)	201 (54.03)	110 (29.57)	-
PLA2R ab (RU/mL)	28.22 (3.50, 96.38)	1.41 (1.08, 2.24)	240.10 (74.17, 369.18)	78.59 (42.95, 158.81)	4.57 (1.65, 9.47)	<0.01
Female (%)	135 (36.29)	22 (40.74)	4 (57.14)	71 (35.32)	38 (34.55)	0.57
Age (y)	56.00 (43.75, 64.00]	53.50 (38.75, 64.00)	53.00 (44.00, 63.50)	57.00 (46.00, 65.00)	54.00 (41.50, 62.00)	0.50
Microscopic Hematuria (%)	174 (46.77)	22 (40.74)	4 (57.14)	96 (47.76)	52 (47.27)	0.76
Urine protein (g/24 h)	5.71 (4.11, 8.25)	5.39 (3.97, 6.79)	6.84 (4.45, 8.19)	6.38 (4.33, 9.06)	5.09 (3.64, 7.37)	0.01
Albumin (g/L)	21 (17, 26)	23.5 (17, 28)	14 (13, 21)	21 (16, 25)	23.5 (18, 29)	<0.01
eGFR-EPI (mL/min × 1.73 m^2^)	99.13 (78.61, 113.92)	101.88 (87.33, 113.94)	99.71 (85.91, 119.00)	96.07 (74.60, 113.58)	99.62 (86.55, 112.13)	0.48
Uric acid (umol/L)	350.5 (295.5, 401.3)	352.0 (297.0, 391.0)	319.0 (302.5, 363.5)	350.0 (293.0, 403.0)	352.0 (300.5, 401.5)	0.87
Hypertension (%)	170 (45.70)	12 (22.22)	3 (42.86)	101 (50.25)	54 (49.09)	<0.01
Diabetes Mellitus (%)	52 (13.98)	7 (12.96)	1 (14.29)	31 (15.42)	13 (11.82)	0.84
Treatment (%)						0.1
Pred + CTX	123 (33.06)	16 (29.63)	3 (42.86)	73 (36.32)	31 (28.18)	
pred + CSA	138 (37.10)	19 (35.19)	3 (42.86)	80 (39.80)	36 (32.73)	
ACEI/ARB	95 (25.54)	19 (35.19)	1 (14.29)	38 (18.91)	37 (33.64)	
RTX	16 (4.30)	0 (0.00)	0 (0.00)	10 (4.98)	6 (5.45)	
Secondary causes (%)	43 (11.56)	13 (24.07)	1 (14.29)	19 (9.45)	10 (9.09)	0.02
Pathology stage (%)						<0.01
I	77 (20.70)	21 (38.89)	0 (0.00)	31 (15.42)	25 (22.73)	
II	246 (66.13)	24 (44.44)	6 (85.71)	143 (71.14)	73 (66.36)	
III + IV	49 (13.17)	9 (16.67)	1 (14.29)	27 (13.43)	12 (10.91)	
Tubulo-interstitial lesions ≥ 25(%)	8 (2.15)	0 (0.00)	0 (0.00)	6 (2.99)	2 (1.82)	0.56
Prognosis * (N)	329	41	6	182	100	
Follow-up time (m)	79.20 (48.70, 97.40)	84.00 (51.77, 98.43)	85.23 (60, 97.40)	74.55 (28.65, 80.13)	84.22 (44.63, 106.75)	0.22
Death (%)	13 (3.95)	2 (4.88)	1 (16.67)	8 (4.40)	2 (2.00)	0.29
ESRD (%)	38 (11.55)	3 (7.32)	2 (33.33)	27 (14.84)	6 (6.00)	0.04
Tumor (%)	12 (3.65)	0 (0.00)	0 (0.00)	6 (3.30)	6 (6.00)	0.33
Thrombosis (%)	25 (7.60)	3 (7.32)	1 (16.67)	14 (7.69)	7 (7.00)	0.86
Decrease in eGFR ≥ 30% (%)	73 (22.19)	4 (9.76)	4 (66.67)	47 (25.82)	18 (18.00)	<0.01
6m remission (%)	183 (55.62)	30 (73.17)	1 (16.67)	87 (47.80)	65 (65.00)	<0.01
24m remission (%)	281 (85.41)	39 (95.12)	5 (83.33)	145 (79.67)	92 (92.00)	0.01

Continuous variables presented as mean± SD or median (IQR); Abbreviations: pred + CTX: prednisone combined with cyclophosphamide; pred + CSA: prednisone combined with cyclosporine. ACEI/ARB: Angiotensin-Converting Enzyme Inhibitors/Aldosterone Receptor Blockers; RTX: Rituximab. * Prognosis: patients with secondary causes were removed to reduce confounders in prognosis analysis.

**Table 2:** Univariate and multivariate Cox progression analysis of baseline variables for renal progression in primary PMNs.

	Univariate Analysis		Multivariate Analysis	
	HR: 95% CI	*p* Value	HR: 95% CI	*p* Value
Male (%)	1.18 (0.73–1.92)	0.49		
Age (y)	1.06 (1.04–1.08)	<0.01	1.06 (1.03–1.08)	<0.01
Microscopic Hematuria (%)	0.79 (0.49–1.25)	0.31		
Urine protein (g/24 h)	1.02 (0.96–1.09)	0.48		
Albumin (g/L)	0.97 (0.94–1.01)	0.11		
eGFR-EPI (mL/min × 1.73 m^2^)	0.98 (0.97–0.99)	<0.01	1.004 (0.99–1.02)	0.50
Uric acid (umol/L)	1.001 (0.999–1.004)	0.32		
Hypertension (%)	1.75 (1.10–2.80)	0.02	0.92 (0.55–1.57)	0.77
Diabetes Mellitus (%)	2.89 (1.71–4.90)	<0.01	2.35 (1.35–4.09)	<0.01
Immunosuppressive therapy (%)	2.73 (1.36–5.48)	0.01		
Pathology stage (%)				
I	reference			
II	1.32 (0.72–2.40)	0.37		
III + IV	1.37 (0.62–3.03)	0.44		
Tubulo-interstitial lesions ≥ 25 (%)	2.98 (1.08–8.18)	0.03	2.34 (0.76–7.19)	0.14
Serum PLA2R ab and glomerular PLA2R staining				
SAb−/GAg−	reference		reference	
SAb+/GAg−	9.13 (2.28–36.62)	<0.01	9.17 (2.26–37.16)	<0.01
SAb+/GAg+	3.26 (1.17–9.07)	0.02	2.32 (0.83–6.53)	0.11
SAb−/GAg+	1.92 (0.65–5.67)	0.24	1.53 (0.51–4.60)	0.45

**Table 3:** Univariate and multivariate logistic progression analysis of baseline variables for remission within 6 months in primary PMNs.

	Univariate Analysis		Multivariate Analysis	
	OR: 95% CI	*p* Value	OR: 95% CI	*p* Value
Male (%)	1.20 (0.76–1.87)	0.43		
Age (y)	0.997 (0.98–1.01)	0.73		
Microscopic Hematuria (%)	0.95 (0.61–1.46)	0.81		
Urine protein (g/24 h)	0.94 (0.89–0.99)	0.046	0.92 (0.85–0.98)	0.02
Albumin (g/L)	1.03 (0.99–1.06)	0.11		
eGFR-EPI (mL/min × 1.73m^2^)	0.999 (0.99–1.01)	0.92		
Uric acid (umol/L)	0.999 (0.997–1.002)	0.90		
Hypertension (%)	0.84 (0.55–1.31)	0.44		
Diabetes Mellitus (%)	0.41 (0.22–0.79)	0.01	0.47 (0.24–0.94)	0.03
Immunosuppressive therapy (%)	1.81 (1.01–2.98)	0.02	3.19 (1.78–5.73)	<0.01
Pathology stage (%)				
I	reference			
II	0.78 (0.45–1.35)	0.38		
III + IV	0.87 (0.41–1.84)	0.71		
Tubulo-interstitial lesions ≥ 25 (%)	0.59 (0.13–2.69)	0.50		
Serum PLA2R ab and glomerular PLA2R staining				
SAb−/GAg−	reference		reference	
SAb+/GAg−	0.07 (0.01–0.70)	0.02	0.06 (0.01–0.56)	0.01
SAb+/GAg+	0.34 (0.16–0.71)	<0.01	0.30 (0.14–0.66)	<0.01
SAb−/GAg+	0.68 (0.31–1.52)	0.35	0.70 (0.30–1.61)	0.40

**Table 4:** Comparison of response to immunosuppressive therapy among four subgroups.

CTX	SAb+/GAg+	SAb−/GAg+	SAb−/GAg−	SAb+/GAg−
N	63	29	12	3
6 m remission (%)	30 (47.62)	14 (48.28)	8 (66.67)	0 (0)
OR	reference	1.03 (0.43–2.48)	2.20 (0.60–8.06)	-
OR adjusted ^a^	reference	0.94 (0.38–2.33)	2.10 (0.56–7.88)	-
CSA	SAb+/GAg+	SAb−/GAg+	SAb−/GAg−	SAb+/GAg−
N	76	31	16	2
6 m remission (%)	44 (57.89)	30 (96.77)	12 (75)	1 (50)
OR	reference	21.82 (2.83–168.42) **	2.18 (0.64–7.39)	0.73 (0.04–12.07)
OR adjusted ^a^	reference	16.77 (2.10–134.14) *	2.21 (0.59–8.27)	0.25 (0.01–4.56)
ACEI/ARB	SAb+/GAg+	SAb−/GAg+	SAb−/GAg−	SAb+/GAg−
N	35	35	13	1
6 m remission (%)	10 (28.57)	18 (51.43)	10 (76.92)	0 (0)
OR	reference	2.65 (0.99–7.11)	8.33 (1.89–36.76) *	-
OR adjusted ^a^	reference	2.50 (0.90–6.99)	12.25 (2.48–60.53) **	-

Abbreviations: CTX: cyclophosphamide; CSA: Cyclosporine; ACEI/ARB: Angiotensin-Converting Enzyme Inhibitors/Aldosterone Receptor Blockers. ^a^: adjusted by age, sex, urine protein, and eGFR. * *p* < 0.05; ** *p* < 0.01.

## Data Availability

The datasets used in this study are available from the corresponding author upon reasonable request.
